# Transapical off-pump mitral valve repair following prior mitral valve surgery: A case report

**DOI:** 10.1097/MD.0000000000026148

**Published:** 2021-05-28

**Authors:** Hermann Blessberger, Joerg Kellermair, Juergen Kammler, Clemens Steinwender, Andreas F. Zierer

**Affiliations:** a Department of Cardiology, Kepler University Hospital, Medical Faculty, Johannes Kepler University, Linz; b Department of Internal Medicine II, Paracelsus Medical University, Salzburg; c Department of Cardiothoracic and Vascular Surgery, Kepler University Hospital, Medical Faculty, Johannes Kepler University, Linz, Austria.

**Keywords:** minimally invasive repair, mitral regurgitation, NeoChord device, redo procedure

## Abstract

**Rationale:**

Redo surgeries after mitral valve repair are technically demanding. Procedures applying the NeoChord device (NeoChord Inc, St. Louis Park, MN) have proven to be safe and feasible in selected patients requiring mitral valve repair due to a leaflet prolapse or flail. However, its use for redo procedures after conventional surgical repair has not been well established yet.

**Patient concerns:**

We report the case of a 57-year-old man who presented with dyspnea upon exertion. The patient had undergone a minimally invasive surgical mitral valve repair because of a flail leaflet of the segments segment 2 of the posterior mitral valve leaflet (P_2_)/segment 3 of the posterior mitral valve leaflet (P_3_) 4 years before.

**Diagnoses:**

Transesophageal echocardiography identified a relapse of severe mitral valve regurgitation. The recurring regurgitant jet was caused by a flail leaflet due to newly ruptured native chords.

**Interventions:**

After discussion in an interdisciplinary heart team, we performed a minimally invasive off-pump redo procedure applying the NeoChord device under three-dimensional transesophageal echocardiographic guidance.

**Outcomes:**

The echocardiographic result with only trivial residual mitral regurgitation as well as the further clinical course of the patient were favorable.

**Lessons:**

As redo surgery after minimally invasive mitral valve repair is challenging, the NeoChord device represents a novel treatment option that does not require cardiopulmonary bypass.

## 1 Introduction

The probability of recurrent regurgitation after prior surgical mitral valve repair depends on the underlying pathology as well as the completeness and mode of the repair.^[1,2]^ In patients with bi-leaflet repair, recurrent mitral regurgitation was reported in 17% or even higher.^[2]^ Despite a clinical benefit,^[3]^ the number of redo surgeries in patients with recurrent mitral regurgitation after surgical repair is rather low,^[1,2]^ as the problem can only be solved by a second surgical repair or a valve replacement requiring another on-pump procedure with sternotomy. Besides these surgical procedures in the strict sense, minimally invasive techniques for this indication are emerging. In our center, we routinely perform a video-assisted minimally invasive approach through a right mini thoracotomy for the initial mitral and tricuspid valve repair. This access can also be very cumbersome in redo cases due to scarring after the first operation. We applied a new technology to overcome these problems.^[4]^ We present the following case in accordance with the CARE reporting guidelines for case reports.^[5]^ Local ethics committee approval for writing this case report was sought and granted. The patient has provided written informed consent for publication of this case report.

## 2 Case report

**Figure 1:**
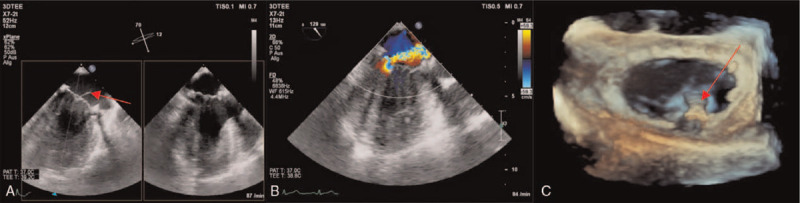
TEE of mitral valve after surgical repair with recurrent regurgitation before intervention. Panel A: Biplane view of the mitral valve. Red arrow indicates a flail leaflet. Panel B: Long axis view of the mitral valve with an anteriorly directed, eccentric regurgitation jet. Panel C: 3D surgeon's view of the mitral valve with annuloplasty ring. Red arrow points to the NeoChord device that is located posteriorly between segments P_2_ and P_3_ and passing through the valve from the left ventricle into the left atrium. 3D = three dimensional, TEE = transesophageal echocardiography.

**Figure 2:**
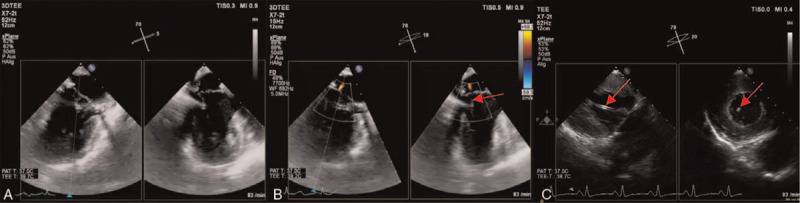
TEE of the mitral valve after NeoChord implantation. Panel A: Biplane view without a residual prolapse or flail leaflet. Panel B: Biplane view with Doppler color flow imaging revealing only a trivial residual mitral regurgitation after implant. Red arrow points to neo-chord that was attached to the posterior mitral leaflet. Panel C: Transgastric biplane long and short axis views of the left ventricle. Red arrows point to the echo-dense neo-chordae.

We report the case of a 57-year-old male patient with recurring significant mitral valve regurgitation after repair of a flail leaflet 4 years earlier. During the first procedure, a Carpentier Edwards classic mitral annuloplasty ring (38 mm) with duraflo treatment (Edwards Life sciences; Irvine, CA, US) and 1 artificial gore chord (Gore-Tex; W.L. Gore & Associates Inc., Flagstaff, AZ, USA) had been implanted after triangular resection of the P2/P3 segment with no residual mitral regurgitation. The patient did well until he was readmitted 4 years later because of mild to moderate dyspnea upon exertion. The transthoracic echocardiogram showed severe mitral regurgitation. Subsequent transesophageal echocardiography (TEE) found a recurring flail leaflet of the P_2_/P_3_ segments (Fig. 1A and B). This was caused by rupture of other native chordae, whereas the artificial gore chord was still in situ without dysfunction. Left ventricular function was normal and there was no evidence of pulmonary hypertension. The NT-pro BNP level was within normal range (150 ng/L), and significant coronary artery stenoses were ruled out by angiography. The patient was discussed in the institutional interdisciplinary heart team. We decided to go for an off-pump minimally invasive mitral valve repair using the NeoChord system (NeoChord Inc., St. Louis Park, MN, investigational device exemption approval by the U.S. Food and Drug Administration). The prior implantation of an annuloplasty ring favored the NeoChord approach because the normally sized mitral annulus ensured a sufficient amount of leaflet tissue to cover the mitral orifice and create an adequate surface of coaptation. The visual guidance during the NeoChord procedure solely relies on ultrasound (Fig. 1C). The patient underwent NeoChord implantation as described before.^[4,6,7]^ In brief, the mitral leaflet was grasped with the NeoChord device under three-dimensional TEE guidance after a posterolateral transapical access.^[8]^ Gore-Tex CV-4 sutures (Gore-Tex; W.L. Gore & Associates Inc., Flagstaff, AZ, USA) were deployed along the free edge of the flailing segment. The artificial chords were passed through the ventricular access site, and the length and tension were assessed under TEE guidance, obtaining full mitral competence. The artificial chords were then knotted down on an additional small Teflon felt that served as an abutment.^[4,6]^ Despite a favorable result after the first chord implantation in segment P_2_, we decided to add a second chord in segment P_3_. This was done to reduce the shear stress by splitting it among several different chordae.^[4,9]^ After proper tensioning and fixation of the neo-chordae on the epicardial surface, only a trivial mitral valve regurgitation remained (Fig. 2). The postoperative course of the patient was uneventful. He was transferred to the regular ward the day after the procedure and left hospital on postoperative day 10. The patient was clinically symptom-free at a visit 16 months after the procedure. Follow-up echocardiography showed a good long-term result without any mitral regurgitation.

## 3 Discussion

The novel NeoChord system allows a minimally invasive off-pump mitral valve repair with neo-chordae in patients requiring mitral valve repair due to a leaflet prolapse or flail. In a large European registry as well as in a recent meta-analysis, it has been shown that mitral valve repair with the NeoChord system was safe, feasible and durable with a low perioperative morbidity.^[10,11]^ As it is a “ringless” procedure, a certain length of the leaflets has to be ensured to enable the grasping with the NeoChord device and to allow for the proper coaptation of the leaflets after fixation of the neo-chordae. This limitation must be considered when selecting patients. In this regard, the echocardiographically assessed “leaflet-to-annulus index (LAI)” can help to estimate feasibility and success of the procedure.^[12]^ In case of a dilated annulus, tensioning of the mitral valve leaflets by the neo-chordae would cause a leaflet restriction resulting in a co-adaptation defect and remarkable residual mitral regurgitation. Furthermore, the more centrally the prolapse is located, the easier it can be grasped as opposed to more medially or laterally located lesions. Despite being successfully used in initial mitral valve repairs in suitable subjects, the use of the NeoChord device for redo procedures after surgical mitral valve repair is not well established yet. Our patient was well suited for the NeoChord procedure because after prior annuloplasty ring implantation both leaflets were still long enough to ensure sufficient coaptation and to avoid leaflet restriction. However, the grasping of the leaflets could have been hindered by the previously implanted ring, as the implantation system could have gotten stuck in it. This is especially important to consider, as grasping of the leaflets is usually accomplished by moving the chaws of the NeoChord device towards the native mitral annulus to ensure a proper grip. However, this potential obstacle did not arise during implantation, and after 16 months no residual mitral regurgitation on the transthoracic echocardiogram could be detected. We conclude that in selected patients with recurrent mitral valve regurgitation after mitral valve repair, a minimally invasive off-pump redo using the NeoChord system is a feasible option.

## Author contributions

**Conceptualization:** Hermann Blessberger, Juergen Kammler, Clemens Steinwender, Andreas F. Zierer.

**Project administration:** Hermann Blessberger.

**Visualization:** Hermann Blessberger, Joerg Kellermair, Juergen Kammler.

**Writing – original draft:** Hermann Blessberger, Clemens Steinwender, Andreas F. Zierer.

**Writing – review & editing:** Joerg Kellermair, Juergen Kammler.
